# Pain assessment of traumatic brain injury victims using the Brazilian version of the Behavioral Pain Scale

**DOI:** 10.5935/0103-507X.20180009

**Published:** 2018

**Authors:** Caíque Jordan Nunes Ribeiro, Andra Carla Santos de Araújo, Saulo Barreto Brito, Daniele Vieira Dantas, Mariangela da Silva Nunes, José Antonio Barreto Alves, Maria do Carmo de Oliveira Ribeiro

**Affiliations:** 1 Postgraduate Program in Health Sciences, Universidade Federal de Sergipe - Aracaju (SE), Brazil.; 2 Nursing Department, Universidade Federal de Sergipe - Aracaju (SE), Brazil.; 3 Postgraduate Program in Nursing Universidade Federal de Sergipe - Aracaju (SE), Brazil.

**Keywords:** Nociceptive pain, Pain measurement, Deep sedation, Craniocerebral trauma, Intensive care units

## Abstract

**Objective:**

To evaluate the validity and reliability of the Brazilian version of the Behavioral Pain Scale (BPS-Br) in victims of traumatic brain injury.

**Methods:**

Observational prospective study with paired and repeated measures conducted at two intensive care units (clinical and surgical) of a large general hospital. The convenience sample consisted of adult victims of moderate or severe penetrating or blunt craniocerebral trauma who were sedated and mechanically ventilated. A total of 432 paired observations were performed by independent evaluators simultaneously, prior to eye cleaning, during eye cleaning, during tracheal aspiration and after tracheal aspiration. Sociodemographic, clinical, trauma-related, sedoanalgesia and physiological parameter data (heart rate, systolic and diastolic blood pressure) were collected. The discriminant validity was tested using the Friedman and Wilcoxon paired tests. The intraclass correlation coefficient and Cohen's Kappa coefficient were used to evaluate the reliability. The Spearman correlation test was used to test the association between clinical variables and BPS-Br scores during tracheal aspiration.

**Results:**

There was a significant increase in the physiological parameters during tracheal aspiration, but without correlation with the BPS-Br scores. Pain was significantly more intense during tracheal aspiration (p < 0.005). Satisfactory interobserver agreement was found, with an intraclass correlation coefficient of 0.95 (0.90 - 0.98) and Kappa coefficient of 0.70.

**Conclusion:**

Brazilian version of the Behavioral Pain Scale scores increased during tracheal aspiration. The Brazilian version of the scale was valid and reliable for pain assessment of traumatic brain injury victims undergoing tracheal aspiration.

## INTRODUCTION

The inability to report pain does not exclude the possibility of its existence, given it is an individual, subjective and multidimensional experience related to actual or potential damage.^(1)^

Patients in critical care units (ICUs) are routinely submitted to procedures performed by multiprofessional teams to maintain their basic organ functions. Some interventions are characterized as using nociceptive stimuli that, although necessary, impair comfort and cause pain.^(2)^ Mobilization, wound care, tracheal aspiration (TA), and arterial puncture are some examples of procedures cited as painful by patients interviewed after discharge from the ICU.^(3,4)^

The pain management, restlessness and *delirium* in patient-centered ICUs is a current concern in recent international guidelines, with the objective of ensuring better outcomes related to mortality, physiological complications, length of hospital stay and mechanical ventilation time.^(5,6)^ Despite the expansion of such knowledge, appropriate pain management in critically ill patients is still a challenge.

Sedation, a decreased level of consciousness, intubation and mechanical ventilation are some of the factors that make pain assessment via self-report unfeasible,^(1)^ requiring the use of specific instruments in these situations. Studies on observational tools for pain assessment in patients unable to self-report have been continuously developed at the international level.^(7-9)^

The Behavioral Pain Scale (BPS) and the Critical-Care Pain Observation Tool (CPOT) are scales based on pain-related behaviors, have evidence of validity and reliability and are recommended by guidelines and protocols for pain management in the ICU.^(5,6)^ In Brazil, only the BPS has been culturally adapted to Brazilian Portuguese, validated with intubated patients from a general ICU^(10,11)^ and in the postoperative period of heart surgeries.^(12)^ However, additional validations with different patient populations are fundamental because of the pathophysiological particularities of each situation.

Although pain is an experience often associated with trauma, pain underestimation and undertreatment (oligoanalgesia) are constant phenomena among care teams.^(13,14)^ Due to the severity of the condition and the association with multiple trauma, the situation becomes more worrisome in regard to victims of traumatic brain injury (TBI), as there are few studies related to this topic.^(15,16)^ TBI is a serious public health problem that leads to disability, impaired quality of life and chronic pain.^(17)^ Thus, the objective of this study was to evaluate the validity and reliability of the Brazilian version of the BPS (BPS-Br) in TBI victims.

## METHODS

We conducted an observational, prospective study with repeated and paired measures in a surgical ICU and a clinical ICU of a large general hospital in Aracaju, Sergipe (SE), Brazil, from September 2015 to June 2016.

Sampling was non-probabilistic by convenience, with an estimated sample size of approximately 25 - 30 patients. The sample size calculation was based on Cronbach's alpha coefficient with precision of 0.90 ± 0.05 for a scale with three subscales, according to previous studies.^(12,18)^

Critically ill patients over 18 years old, victims of moderate or severe penetrating or blunt TBI, sedated and mechanically ventilated for more than 48 hours were considered.

Quadriplegia, underlying neurological disease, use of neuromuscular blockers, suspected brain death, hemodynamic instability and use of resuscitation measures were considered exclusion criteria because they interfered with the manifestation of pain-related behaviors and were used in previous studies.^(12,18,19)^ Patients who had scheduled extubation, were discharged to the ward, or died before the second assessment were also excluded.

Sociodemographic and clinical data and data related to the trauma incident, prescribed analgesics and sedation were obtained from medical records and in interviews with family members of the participants.

The Acute Physiology and Chronic Health Evaluation (APACHE II) score was used to describe the severity of the clinical condition in the first 48 hours of ICU stay. The level of sedation was measured using the Ramsay and Richmond Agitation-Sedation Scale (RASS) scores prior to the pain assessments using the BPS-Br.

**Figure 1:**
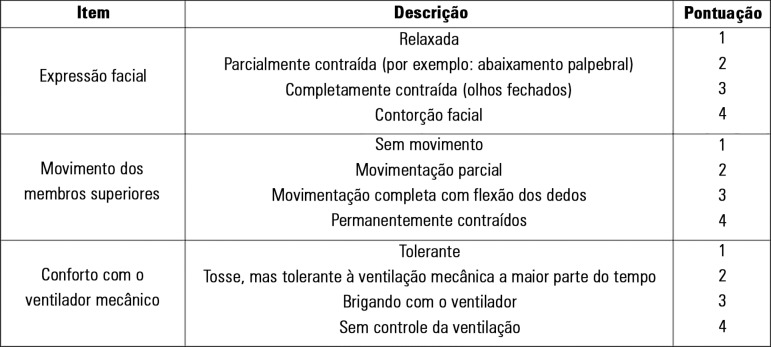
Brazilian version of the Behavioral Pain Scale (BPS-Br).

Source: Azevedo-Santos IF, Alves IG, Badauê-Passos D, Santana-Filho VJ, DeSantana JM. Psychometric analysis of Behavioral Pain Scale Brazilian Version in sedated and mechanically ventilated adult patients: a preliminary study. Pain Pract. 2016;16(4):451-8.^(11)^

The BPS version used in this study was adapted by Azevedo-Santos et al.^(11)^ (Figure 1). The BPS-Br has three subscales that are scored from 1 to 4, for total scores ranging from 3 (no pain) to 12 (inadmissible pain). Total scores > 3 indicate the presence of pain and ≥ 5 indicate significant pain.^(20)^

The physiological parameters heart rate (HR), systolic blood pressure (SBP) and diastolic blood pressure (DBP) were recorded during the collection of BPS-Br scores. Health professionals constantly associate the fluctuation of these parameters with the presence of pain.

The collection team consisted of three nursing students and one medical student. The study assistants received theoretical training, provided by the principal investigator, on general concepts of pain assessment and management, physiological and behavioral indicators of pain in critically ill patients, as well as on the BPS-Br. The pilot test was performed with three patients for practical training and adjustment of the collection form. The data from the pilot study were discarded and were not part of the final analysis.

The BPS-Br was conducted in a paired manner, simultaneously by two independent observers, and there was no communication between them during the assessment. This procedure was repeated on a second occasion, during a different shift from the first collection, in order to obtain a larger number of observations. BPS-Br scores were collected 1 minute prior to eye cleaning (EC) during EC, during TA and 10 minutes after TA. The baseline or reference BPS-Br score was considered the score obtained when the patient was resting, that is, before the non-nociceptive stimulus.

### Statistical analysis

All data were analyzed descriptively. Numerical variables were expressed as the mean ± standard error of the mean, and categorical variables were expressed in absolute and relative frequencies. The symmetry of the distribution was tested by the Shapiro-Wilks test. The Spearman correlation test was performed to examine the association between physiological parameters, APACHE II scores, Ramsay scores, RASS scores, and total BPS-Br scores. The discriminant validity was evaluated using the non-parametric Friedman test for the four distinct assessment times, and the non-parametric Wilcoxon test for post hoc pairwise comparisons. Reliability was assessed through interobserver agreement, with calculation of the intraclass correlation coefficient (ICC) and Cohen's Kappa coefficient.^(21)^

This study was approved by the Ethics Committee of the *Universidade Federal de Sergipe* (Opinion 903,798) and followed the recommendations of the Declaration of Helsinki and the National Health Council Resolution 466/2012. An informed consent form was signed by the participants' legal guardian because the participants were unable to make decisions. Tracheal aspiration was performed exclusively by professionals from the physiotherapy care team according to the needs of the patients. No additional procedures were performed for the benefit of this study.

## RESULTS

A total of 37 patients were recruited to compose the sample. Ten were excluded because they were extubated, discharged to the ward, or died before the second assessment. Thus, we obtained a final sample of 27 patients, for a total of 432 observations (27 patients × 2 observers × 4 observation times × 2 assessments).

In our study, severe TBI prevailed (88.9%), caused by blunt trauma mechanisms due to automobile collisions involving motorcycles (74.1%), in which the victims did not use the recommended safety device (66.7%) (Table 1).

**Table 1:** Clinical and sociodemographic data

Variables	Specification
Sex	
Male	25 (92.6)
Female	2 (7.4)
Age	39.3 ± 2.7
Education in full years of study	4.1 ± 0.7
Marital status	
With partner	14 (51.9)
Without partner	13 (48.1)
Ethnicity	
Non-white	18 (66.7)
White	9 (33.3)
Place of residence	
Interior of the state	19 (70.4)
Metropolitan region	8 (29.6)
Days of hospitalization	7.0 ± 0.6
Days of hospitalization in ICU	4.3 ± 0.6
Days on mechanical ventilation	6.9 ± 0.7
Inpatient ICU	
Clinical	18 (66.7)
Surgical	9 (33.3)
APACHE II score	15.7 ± 1.2
Initial GCS score	7.1 ± 0.6

ICU - intensive care unit; APACHE - Acute Physiology and Chronic Health Evaluation; GCS - Glasgow Coma Scale. Values expressed as N (%) or mean ± standard error.

Only one record of pain, made by the doctor and physiotherapist, was found in the chart. Midazolam and fentanyl were the drugs used to make the standard sedoanalgesia solutions at the institution. Patients were deeply sedated in both assessments (Ramsay: 5.6 ± 0.1 and 5.2 ± 0.2, RASS = -3.9 ± 0.3 and -3.7 ± 0.4). Simple analgesics, non-steroidal anti-inflammatories and other opioids were prescribed in an irregular manner. Infusion of the sedoanalgesia solution was active during most assessments (Table 2).

**Table 2:** Sedation and analgesia

Variables	Assessment		Infusion speed (mL/hr)
	First n (%)	Second n (%)	
Sedatives prescribed			
Midazolam	23 (85.2)	20 (74.1)	15.3 ± 3.2
Propofol	1 (3.7)	-	-
None	4 (14.8)	7 (25.9)	-
Analgesics prescribed			
Dipyrone	26 (96.3)	25 (92.6)	-
Fentanyl	23 (85.2)	24 (88.9)	14.1 ± 1.5
Paracetamol	10 (37.0)	12 (44.4)	-
Methadone	4 (14.8)	2 (7.4)	-
Morphine	1 (3.7)	-	-
None	-	2 (7.4)	-
Active infusion of sedoanalgesia solution			
Yes	18 (66.7)	19 (70.4)	-
No	9 (33.3)	8 (29.6)	-

**Figure 2:**
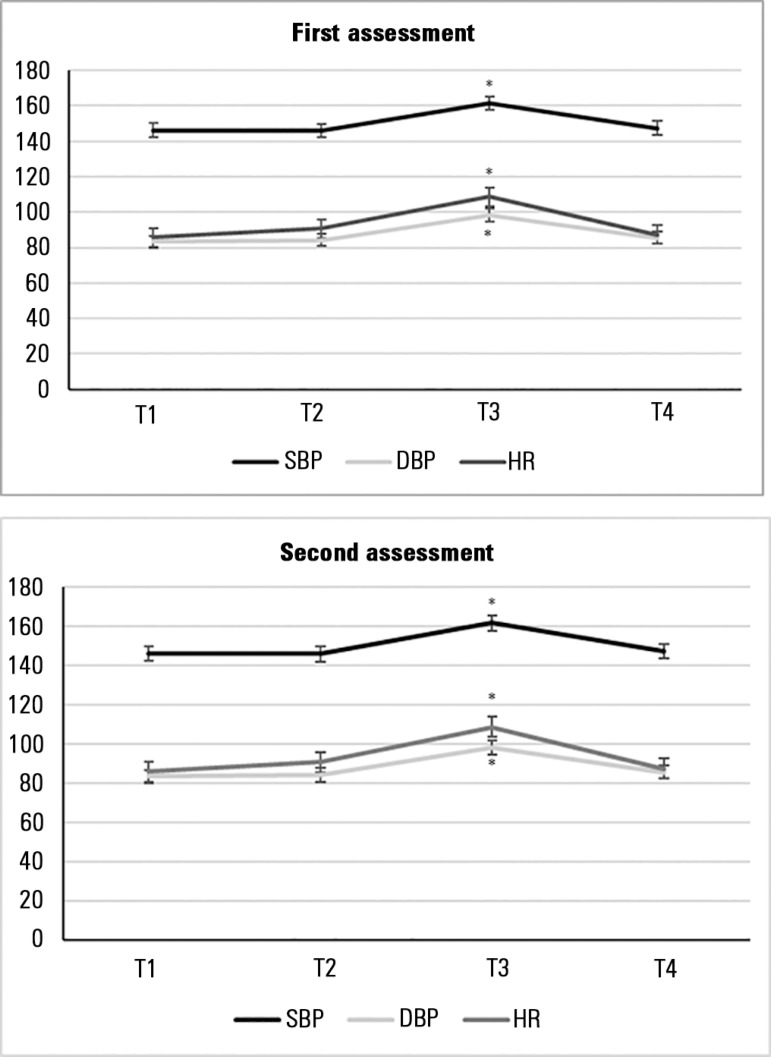
Fluctuation in physiological parameters during the assessments. T1 - prior to eye cleaning; T2 - during eye cleaning; T3 - during tracheal aspiration; T4 - after tracheal aspiration; SBP - systolic blood pressure; DBP - diastolic blood pressure; HR - heart rate. * p ≤ 0.005 Friedman test and *post hoc* Wilcoxon paired test.

For the physiological parameters the mean SBP, DBP and HR increased significantly during TA, returning to baseline values 10 minutes after the nociceptive stimulus (Figure 2).

**Figure 3:**
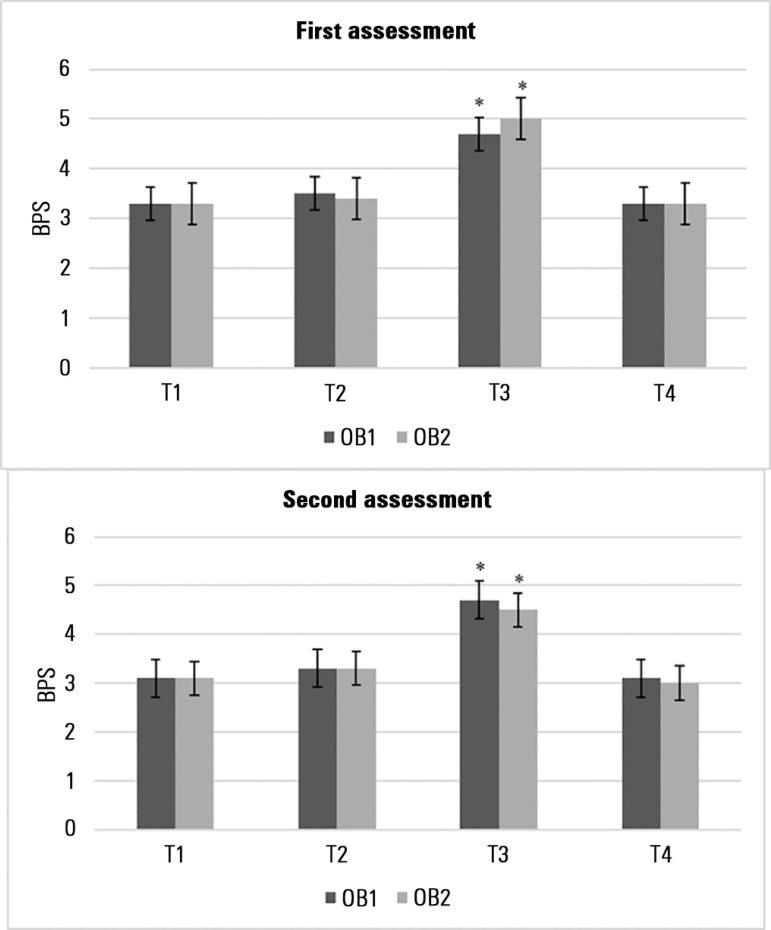
Behavioral Pain Scale total scores according to the different observation times. BPS - Behavioral Pain Scale; T1 - prior to eye cleaning; T2 - during eye cleaning; T3 - during tracheal aspiration; T4 - after tracheal aspiration; OB1 - observer 1; OB2 - observer 2. * p < 0.0001 Friedman test and *post hoc* Wilcoxon paired test.

The BPS-Br scores increased significantly during TA in both assessments, which did not occur during EC. Similar values were obtained during rest and the retest observation, confirming the discriminant validity of the BPS-Br score increasing during painful procedures (Figure 3).

There was no significant correlation between clinical parameters, physiological parameters and total BPS-Br scores during TA. Only the Ramsay and RASS scores correlated with the BPS-Br scores recorded by observer 1 in the first assessment (Table 3).

**Table 3:** Correlations between clinical variables and Behavioral Pain Scale scores during tracheal aspiration in the first assessment

Variables	Total BPS-Br score during TA			
	Observer 1		Observer 2	
	rho	p value	rho	p value
APACHE II	-0.17	0.39	-0.80	0.69
Ramsay	-0.48	0.01*	-0.30	0.13
RASS	0.45	0.02*	0.35	0.07
Devices in use	0.19	0.35	0.07	0.75
SBP	0.14	0.48	0.14	0.50
DBP	0.26	0.20	0.31	0.11
HR	-0.16	0.41	0.09	0.64

BPS - Behavioral Pain Scale; TA - tracheal aspiration; APACHE - Acute Physiology and Chronic Health Evaluation; RASS - Richmond Agitation-Sedation Scale; SBP - systolic blood pressure; DBP - diastolic blood pressure; HR - heart rate. Spearman correlation.

Regarding reliability, ICC values of 0.95 (95% confidence interval [95%CI]: 0.90 - 0.98) and 0.89 (95%CI: 0.75 - 0.95) and Kappa coefficients of 0.45 and 0.60 were obtained in the first and second assessments, respectively, when comparing the scores attributed by the observers during TA. Among the subscales, upper limb movement (ICC: 0.92 - 1.00, Kappa: 0.70 - 1.00), followed by compliance with mechanical ventilation (ICC: 0.83 - 0.88, Kappa: 0.63 - 0.81) obtained the best agreements (Table 4).

**Table 4:** Reliability analysis of the Behavioral Pain Scale during tracheal aspiration

BPS scores	Interobserver reliability	
	ICC (95%CI)*	Kappa^†^
Facial expression		
Assessment 1	0.93 (0.85 - 0.97)	0.69
Assessment 2	0.60 (0.12 - 0.82)	0.55
Upper limb movement		
Assessment 1	0.92 (0.68 - 0.98)	0.70
Assessment 2	1,00 (-)	1.00
Compliance with mechanical ventilation		
Assessment 1	0.83 (0.63 - 0.92)	0.63
Assessment 2	0.88 (0.74 - 0.95)	0.81
Total		
Assessment 1	0.95 (0.90 - 0.98)	0.45
Assessment 2	0.89 (0.75 - 0.95)	0.60

BPS - Behavioral Pain Scale; ICC - intraclass correlation coefficient; 95% CI - 95% confidence interval.

* ≥ 0.80 ideal

† 0.41 - 0.60 moderate; 0.61 - 0.80 substantial; 0.81 - 1.0 almost perfect.

## DISCUSSION

Pain relief is a fundamental patient right^(22)^ which must be ensured regardless of their level of consciousness. The use of valid and reliable instruments to assess pain is a key step for its proper management.^(23)^ TBI is often associated with multiple trauma and the need for ICU hospitalization. Thus, in addition to the pain associated with trauma, during the resting period, the use of invasive devices and routine procedures may intensify the pain experience.^(18)^

There was no association between the clinical variables and the BPS-Br scores, which reinforces the premise that pain is an individual experience, and its intensity and the consequent suffering are not related to the extent of tissue injury or disease severity.^(1)^ However, careful investigation of the patient's medical history cannot be disregarded in view of its importance in the development of an individualized care plan for pain relief.

Regarding analgesia and sedation, deep sedation regimens still persist in the institution where the study was conducted. Midazolam and fentanyl solutions were the most frequently prescribed. By contrast, there is evidence that the exacerbated use of benzodiazepines is associated with negative patient outcomes.^(24)^

The most recent guidelines on analgesia, sedation and *delirium* recommend that the critical patient's comfort is a priority. Adequate pain management leads to more superficial sedation, with lower doses of benzodiazepines, reduced mechanical ventilation time, lower infection rates and pulmonary complications, and greater patient collaboration.^(25-27)^ It was found that post-hospitalization patients report vivid memories about experiences in the ICU. Therefore, more effective interventions are needed for the relief, treatment and prevention of pain by the multiprofessional care team.^(28)^

The use of simple analgesics, non-steroidal anti-inflammatory drugs and other alternative drugs to opioids was inconsistent and prescribed in an irregular manner, that is, "whenever necessary". In addition, it is inferred that such drugs were not prescribed for analgesic purposes because there is no systematic pain assessment at the study institution. Systematic prescription regimens of these substances confer many benefits, such as decreased opioid use and the subsequent onset of related side effects.^(26)^

Physiological parameters are readily available to intensivists and, culturally, have been used as indicators of the presence of pain. However, several studies have shown that vital signs are not specific indicators for pain assessment in critically ill patients.^(29-31)^ Thus, they should not be used as the only source of pain assessment, but just as initial signs for suspecting its existence and furthering investigation.^(8)^ Disease severity, hemodynamic instability, use of vasoactive drugs, anxiety and fear are some of the factors that may alter these via the activation of mechanisms that involve the organic response of catecholamine cascade activation and stress hormone release.^(26)^

In our study, there was a significant increase in SBP, DBP and HR during TA, but there was no correlation with the BPS-Br scores. There are similar reports in the literature regarding HR, SBP, oxygen saturation and respiratory rate.^(9,29,30)^ However, in Arbor et al.,^(29)^ only the respiratory rate was found to be a potential indicator for TBI victims because of its significantly positive correlation with patient self-report, but this necessitates further investigation. We chose not to include the respiratory rate in our data collection because its significant change is expected given the nature of the TA procedure.

The BPS-Br presented good discriminant validity, as during TA the scores increased significantly. It was not possible to evaluate the validity criterion due to the unavailability of a tool that is recognized as the gold standard for pain measurement in unconscious patients. The total scores during the nociceptive stimulus were lower than those reported in previous studies. Possibly, this finding may be associated with the altered consciousness level in TBI victims, secondary to the mechanisms of primary and secondary neurological injuries, as well as the sedation intensity and regimen adopted by the institution.^(32,33)^

With regard to reliability, satisfactory interobserver agreement results were obtained, especially those related to the ICC. Similar results were found in a clinical validation study by Morete et al.^(10)^ We believe that the standardization of data collection, with exhaustive theoretical and practical training of research assistants, contributed to these results. Interestingly, the facial expression subscale presented the lowest agreement coefficient, in contrast to previous studies in which compliance with mechanical ventilation presented lower agreement.^(11,34)^ The psychometric evaluation of the Chinese version of the BPS presented similar high agreement in the facial expression subscale.^(35)^

Some researchers have reported that TBI victims manifest atypical or unusual pain-related behaviors, which may explain why the change in facial expression scores was not as prominent.^(33)^ Facial expression is the behavioral indicator most easily recognized by health professionals.^(36,37)^ Therefore, this unique characteristic of TBI victims can make pain assessment even more challenging.

Tools such as the BPS should be widely disseminated in Brazilian ICUs. Although the BPS was found to be valid, reliable and easy to use, an intense awareness campaign and training for healthcare professionals is imperative for its use to be consistent, efficient and effective.^(38,39)^ In addition, the development and implementation of protocols and clinical guidelines for care centered on the comfort of critical patients are crucial for humanization and improving the quality of care.^(40)^

The lack of blinding of the investigators regarding the observed procedure was a limitation of this study. The principal investigators, however, were excluded from the data collection phase to reduce the possibility of measurement bias. Another limitation was the impossibility of randomizing the inclusion of patients in the study. However, we increased the number of observations to enhance the power of analysis.

## CONCLUSION

The Brazilian version of the Behavioral Pain Scale is a valid and reliable instrument for assessing pain in traumatic brain injury victims. We suggest that it be incorporated into the routine of Brazilian ICUs and that future studies test its psychometric properties with different painful procedures and its impact on patient outcome after implementation, as well as use it as a measurement instrument in randomized controlled trials.
